# Establishing a prediction model for lateral neck lymph node metastasis in patients with papillary thyroid carcinoma

**DOI:** 10.1038/s41598-018-35551-9

**Published:** 2018-11-26

**Authors:** Shan Jin, Wuyuntu Bao, Yun-Tian Yang, Tala Bai, Yinbao Bai

**Affiliations:** Department of General Surgery, Affiliated Hospital of Inner Mongolia Medical University, Hohhot 010050, Inner Mongolia Autonomous Region, Hohhot, China

## Abstract

This study aimed to establish a model for predicting lateral neck lymph node metastasis in patients with papillary thyroid carcinoma. A total of 106 patients with papillary thyroid carcinoma undergoing initial treatment of neck lymph node dissection (including central and lateral neck lymph nodes) at the thyroid surgery department were enrolled from January 2009 to April 2017. Logistic regression analysis was used to screen the factors influencing lateral neck lymph node metastasis and develop a prediction model. The receiver operating characteristic curve was used to evaluate the predictive power and boundary value of the model for lateral neck lymph node metastasis. Prediction model: Logistic(P) = −5.699 + 0.681 × _(TSH)_ + 0.342 × _(Metastatic rate of central lymph nodes)_ + 1.463 × _(Combined with Hashimoto’s disease)_ + 1.525 × _(Number of tumors)_. When logistic (*P*) was ≥ 0.821, it was predicted that lateral neck lymph node metastasis occurred in patients with papillary thyroid carcinoma. When logistic (*P*) was <0.821, it was predicted that no metastasis was found in the lateral neck lymph node. The prediction accuracy was 78.3%. The model helped in evaluating lateral neck lymph node metastasis in patients with papillary thyroid carcinoma. Also, it had significance in determining reasonable surgical range, reducing unnecessary lateral neck lymph node dissection, and further improving the quality of life of patients.

## Introduction

Thyroid cancer, a common malignant tumor of the endocrine system, has become one of the fastest-growing tumors in the world, with the incidence rate increasing year by year^1^. Papillary thyroid carcinoma, the most common pathological type of thyroid cancer, accounts for more than 90% of tumors, less severe malignancy. After operation and postoperative adjuvant therapy, the prognosis becomes better and long-term survival can be achieved. However, lateral neck lymph node metastasis was found in 20–90% of patients with papillary thyroid carcinoma^2,3^. An important risk factor for higher recurrence rate and lower survival rate in patients with thyroid cancer is neck lymph node metastasis^4,5^, especially for lateral neck lymph node metastasis^6^. Lateral neck lymph node dissection is relatively risky and prone to accidental injury and complications because of the specificity of biological behavior of papillary thyroid carcinoma and the complexity of neck lymph node dissection. Therefore, accurate preoperative evaluation of lateral neck lymph node metastasis helps in determining the surgical range and improving tumor-free survival and quality of life of patients. The application of preoperative ultrasound, computed tomography (CT), magnetic resonance imaging (MRI), fine-needle aspiration biopsy, thyroglobulin (TG) test of puncture eluate, positron emission tomography–CT, and mutation test is still controversial in terms of the accuracy of lateral neck lymph node assessment. Combined application increases the accuracy of evaluation to some extent^7–15^. Therefore, predicting lateral neck lymph node metastasis in patients with papillary thyroid carcinoma needed a more objective and accurate method. In this study, logistic regression model and receiver operating characteristic (ROC) curve were used to analyze the clinicopathological features of patients with papillary thyroid carcinoma, so as to predict lateral neck lymph node metastasis. It helped in determining the surgical range, further improving the quality of life of patients and reducing recurrence.

## Results

### Clinicopathological results

A total of 106 patients with papillary thyroid carcinoma were enrolled, including 25 males and 81 females with the male-to-female ratio of 1:3.24. The age range was 15–70 years, with a median age of 41.4 years. All patients underwent total thyroidectomy, as well as ipsilateral central and lateral neck lymph node dissection. The postoperative pathological results of all patients were obtained. The single-factor analysis was used to analyze clinicopathological data. Patient’s gender; age; tumor location; total number of central lymph node; distribution of gland tumor *in vivo*; serum T3, T4, FT3, FT4, TSH, TGAb, and TPOAb levels; and tumor diameter were not found to be related to lateral neck lymph node metastasis (*P* > 0.05). However, capsular invasion, multifocality, number of tumors, and number of central lymph node metastasis were related to lateral neck lymph node metastasis, and the difference was statistically significant (*P* ≤ 0.05) (see Table 1).

**Table 1:** Single-factor analysis of clinicopathological results in patients with papillary thyroid carcinoma.

Index	Grouping	Lateral neck lymph node metastasis				Total		Statistics	*P* value
		No		Yes					
Age		41.5 ± 12.4		41.4 ± 10.9		40.6 ± 10.5		0.035	0.972
Gender	Male	8	25.8	17	22.7	25	23.6	0.120	0.729
	Female	23	74.2	58	77.3	81	76.4		
Thyroid carcinoma combined with Hashimoto’s disease	No	24	77.4	45	60.0	69	65.1	2.929	0.087**
	Yes	7	22.6	30	40.0	37	34.9		
Tumor location	Left lobe	17	56.7	21	41.2	38	46.9	1.820	0.249
	Right lobe	13	43.3	30	58.8	43	53.1		
Capsular invasion	No	28	90.3	53	70.7	81	76.4	4.702	0.030*
	Yes	3	9.7	22	29.3	25	23.6		
Multifocality	No	25	80.6	44	58.7	69	65.1	4.663	0.031*
	Yes	6	19.4	31	41.3	37	34.9		
Distribution of gland tumor *in vivo*	Upper pole	22	71.0	63	84.0	85	80.2		0.219^#^
	Middle part	1	3.2	2	2.7	3	2.8		
	Lower pole	8	25.8	10	13.3	18	17.0		
T3		1.207 ± 0.230		1.190 ± 0.226		1.218 ± 0.219		0.335	0.738
T4		8.502 ± 1.548		8.429 ± 1.751		8.631 ± 1.660		0.195	0.846
FT3		3.290 ± 0.432		3.320 ± 0.584		3.353 ± 0.536		0.259	0.796
FT4		1.347 ± 0.203		1.503 ± 1.805		1.539 ± 1.800		0.478	0.634
TSH		2.531 ± 1.690		3.253 ± 2.146		2.792 ± 1.377		1.671	0.098**
TGAb		59.811 ± 102.341		122.488 ± 181.474		94.485 ± 143.943		1.692	0.094**
TPOAb		40.94 ± 84.23		40.93 ± 70.45		40.93 ± 74.81		0.001	1.000
Tumor diameter (mm)		15.129 ± 10.837		18.040 ± 11.261		17.507 ± 11.450		1.224	0.224
Number of tumors		1.19 ± 0.40		1.67 ± 0.98		1.62 ± 0.89		2.600	0.011*
Number of central lymph node metastasis		2.58 ± 2.38		4.19 ± 3.51		3.72 ± 3.29		2.333	0.022*
Total number of central lymph node		7.03 ± 4.10		6.91 ± 3.92		6.98 ± 4.08		0.161	0.872
Metastatic rate of central lymph nodes		0.376 ± 0.300		0.638 ± 0.360		0.557 ± 0.363		3.566	0.001*

*Note*: *P < 0.05; **P < 0.10; ^#^Fisher exact probability test was adopted; Normal T3 level: 0.8–2.0 ng/ml; normal T4 level: 5.1–14.1 mμg/dl; normal FT3 level: 2.0–4.4 pg/mL; normal FT4 level: 0.93–1.7 ng/dL; normal TSH level: 0.2–4.2 μIU/m; normal TGAb level: 0–115 IU/ml; normal TPOAb level: 0–34 IU/ml;.

### Factors influencing lateral neck lymph node metastasis in patients with papillary thyroid carcinoma analyzed using logistic regression

Single-factor variables with *P* < 0.10, such as thyroid carcinoma combined with Hashimoto’s disease, capsular invasion, multifocality, number of tumors, TSH and TGAb levels, number of central lymph node metastasis, and metastatic rate of central lymph nodes, were included in the logistic regression model. The results showed that TSH, metastatic rate of central lymph nodes, thyroid carcinoma combined with Hashimoto’s disease, and number of tumors were the independent risk factors for lateral neck metastasis in patients with thyroid carcinoma (*P* ≤ 0.05). TSH increased by 1 μIU/m, and the risk of lateral neck lymph node metastasis increased 0.975 times. The metastasis rate of central lymph nodes increased by 10%, and the risk of lateral neck lymph node metastasis increased 0.407 times. The number of tumors increased by 1, and the risk of lateral neck lymph node metastasis increased 3.595 times. The patients with thyroid carcinoma combined with Hashimoto’s disease, and the risk of lateral neck lymph node metastasis increased 4.139 times (shown in Tables 2 and 3).

**Table 2:** Multivariate logistic regression analysis of lateral neck lymph node metastasis in patients with papillary thyroid carcinoma.

Index	Grouping	*β*	Β standardization	SE	Wald *χ²*	OR	OR 95% CI		*P* value
							Lower limit	Upper limit	
TSH		0.681	1.390	0.248	7.514	1.975	1.214	3.213	0.006
Metastasis rate of central lymph node (10%)		0.342	1.156	0.101	11.399	1.407	1.154	1.716	0.001
Thyroid carcinoma combined with Hashimoto’s disease	No					1			
	Yes	1.463	0.701	0.691	4.486	4.139	1.115	16.725	0.034
Number of tumors		1.525	1.335	0.594	6.584	4.595	1.434	14.729	0.010
Constant term		−5.699		1.514	14.168				0.001

**Table 3:** Assignment table of risk factors of lateral neck lymph node metastasis in patients with papillary thyroid carcinoma.

Variable	Variable assignment specification
Thyroid carcinoma combined with Hashimoto’s disease	Yes = 1; No = 0
Capsular invasion	Yes = 1; No = 0
Multifocality	Yes = 1; No = 0
Lateral neck lymph node metastasis	Yes = 1; No = 0
Metastasis rate of central lymph node	0–10% = 1; 11–20% = 2; 21–30% = 3; 31–40% = 4; 41–50% = 5; 51–60% = 6; 61–70% = 7; 71–80% = 8; 81–90% = 9; 91–100% = 10

### Establishment of prediction model

**Figure 1:**
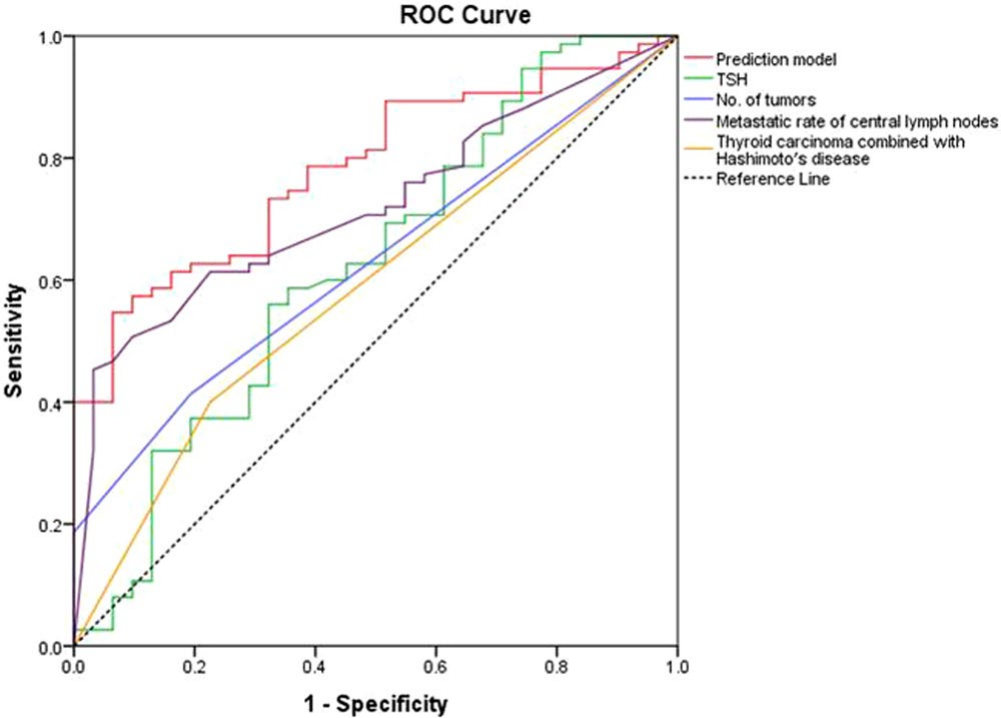
ROC curve for the prediction factors of lateral neck lymph node metastasis in patients with papillary thyroid carcinoma.

Logistic regression was used to screen the risk factors for lateral neck lymph node metastasis in patients with papillary thyroid carcinoma, and a prediction model was established. Prediction model: Logistic(P) = −5.699 + 0.681 × _(TSH)_ + 0.342 × _(Metastatic rate of central lymph nodes)_ + 1.463 × _(Combined with Hashimoto’s disease)_ + 1.525 × _(Number of tumors)_ (TSH by laboratory examination is expressed in μIU/m; Metastatic rate of central lymph nodes = Number of central lymph node metastasis/ Number of dissection central lymph node; Combined with Hashimoto’s disease: Yes = 1, No = 0; Number of tumors: the number of pathologically confirmed; Number of central lymph node metastasis: the number of pathologically confirmed)(detailed model summary please see the supplementary table). ROC curve was used to evaluate the predictive power of various factors for lateral neck lymph node metastasis in patients with papillary thyroid carcinoma. TSH, thyroid carcinoma combined with Hashimoto’s disease, number of tumors, metastasis rate of central lymph node, and prediction model were used as test variables. Then, ROC curve analysis was performed. The boundary value corresponding to the largest cut-off point of Youden’s index was the prediction boundary value of lateral neck lymph node metastasis in patients with papillary thyroid carcinoma (shown in Fig. 1). The results showed that the AUC of prediction model was the largest (0.783; shown in Table 4). The boundary value of prediction model was 0.821, sensitivity 54.7%, specificity 93.5%, Youden’s index 48.2%, positive predictive rate 95.3%, and negative predictive value 46.0%. Other prediction indexes are shown in Table 5. If logistic (*P*) was ≥0.821, it was predicted that lateral neck lymph node metastasis occurred in patients with papillary thyroid carcinoma. If logistic (*P*) was <0.821, it was predicted that no metastasis was found in the lateral neck lymph node. The prediction accuracy was 78.3%.

**Table 4:** AUC of different factors in predicting lateral neck lymph node metastasis in patients with papillary thyroid carcinoma.

Index	AUC	SE	*P* value	AUC 95% CI	
				Lower limit	Upper limit
TSH	0.624	0.063	0.045	0.501	0.746
Metastasis rate of central lymph node	0.721	0.050	<0.001	0.624	0.818
Thyroid carcinoma combined with Hashimoto’s disease	0.587	0.059	0.160	0.471	0.703
Number of tumors	0.628	0.055	0.039	0.520	0.736
Prediction model	0.783	0.044	<0.001	0.696	0.870

**Table 5:** Boundary values of different factors in predicting lateral neck lymph node metastasis in patients with papillary thyroid carcinoma.

Index	Cut-off	TRP	TNP	YI	PPV	NPV
TSH	2.495	56.0%	67.7%	23.7%	79.2%	37.7%
Metastasis rate of central lymph node	79.00%	45.3%	96.8%	42.1%	97.1%	42.3%
Thyroid carcinoma combined with Hashimoto’s disease	0.500	40.0%	77.4%	17.4%	81.1%	34.8%
Number of tumors	1.500	41.3%	80.6%	21.9%	83.8%	36.2%
Prediction model	0.821	54.7%	93.5%	48.2%	95.3%	46.0%

Cut-off, prediction boundary value; TRP, true positive rate (sensitivity); TNP, true negative rate (specificity); YI, Youden’s index; PPV, positive predictive value; NPV, negative predictive value.

## Discussion

Lateral neck lymph node dissection is an important part of the treatment of papillary thyroid carcinoma. Kim *et al*. found that when lateral lymph node metastasis ratio >0.3 and largest lymph node size >3 cm were prognostic factors for papillary thyroid cancer-specific mortality. Stage II patients (eighth edition) with lymph node risk (lateral lymph node metastasis ratio >0.3 or largest lymph node size >3 cm) had a much higher cancer-specific mortality rate (20.9%) than those in the same stage without LN risk (3.2%)^16^. This indicated that an important risk factor for lower survival rate in patients with thyroid cancer is neck lateral lymph node metastasis. In general, central lymph node metastasis is frequent in papillary thyroid carcinoma and is associated with lateral neck metastasis. Because of the specificity of biological behavior of papillary thyroid carcinoma, lateral lymph node skip metastasis (defined as lateral lymph node metastasis without central lymph node metastasis) is also occurred in papillary thyroid carcinoma. Chung *et al*. found that skip metastasis was observed in 7.7% of the papillary thyroid microcarcinoma patients^17^. Lateral neck lymph node dissection is prone to accidental injury and complications because of the complexity of neck dissection. The common complications include, hemorrhage, chyle leakage or lymphatic leak, nerve injury (sympathetic nerve, vagus nerve, phrenic nerve, accessory nerve, hypoglossal nerve, marginal mandibular branch of the facial nerve, brachial plexus, and cutaneous cervical plexus), incisional hydrops, incision infection, skin flap necrosis, facial swelling, and parotid gland leakage. The incidence of hemorrhage was reported as 0.29–2%, chyle leakage as 6–8.3%, sympathetic nerve injury as 5%, vagus nerve injury as 0.14%, phrenic nerve injury as 0.14%, accessory nerve injury as 0.29–6%, hypoglossal nerve injury as 0.29%, injury to the marginal mandibular branch of facial nerve as 0.44%, neuropathic pain of cervical plexus as 48%, shoulder weakness as 27%, incisional hydrops as 2%, incision-related infection as 3–8%, saliva leakage as 0.14%^18–24^ after lateral neck lymph node dissection for treating thyroid carcinoma. This indicated that the complications of lateral neck lymph node dissection could lead to dysfunction and sometimes even affect the quality of life of patients. Therefore, it was necessary to treat each patient seriously and ensure a radical effect while protecting function and reducing the incidence of complications. In addition, the reduction of incidence of complications must be based on the reduction of unnecessary lateral neck lymph node dissection. Hence, the question was how to choose the range of lateral neck lymph node dissection in patients with papillary thyroid carcinoma in clinical practice. The American Thyroid Association (ATA), the National Comprehensive Cancer Network (NCCN), and the Chinese version of guidelines for the treatment of noncentral neck lymph node metastasis (cN1b) in patients with differentiated thyroid carcinoma recommended lateral neck lymph node dissection. A comprehensive evaluation was carried out based on the proportion of central lymph node metastasis; the location, size, and pathological typing of primary lesions of DTC; as well as the status of noncentral neck lymph node metastasis. Selective neck lymph node dissection was performed in part on patients with central neck lymph node metastasis (cN1a)^25–27^. However, the operative indications for lateral neck lymph node dissection in patients with differentiated thyroid carcinoma were not definitely given. Amin *et al*. suggested that clinical examinations and ultrasonography examinations are the most independent and reliable factors to detect lateral neck lymph nodes involvement^28^. Ultrasonography, enhanced CT, and MRI might have some significance in evaluating neck lymph nodes in the treatment of thyroid carcinoma. On the contrary, ultrasonography is related to instrument resolution, operator’s experience, and degree of detail operation. Moreover, assessing parapharyngeal and level VII lymph node is not inadequate. Although CT examination overcomes the disadvantages of ultrasonography, the assessment of neck lymph node micrometastasis is poor, with the problem of contrast allergy. Enhanced MRI is affected by respiratory effects in patients and produces artifacts. Furthermore, its sensitivity is lesser compared with ultrasonography or enhanced CT. Mulla *et al*. analyzed the data of 5587 patients with papillary thyroid carcinoma in 19 studies. They found that the accuracy of preoperative ultrasonography and CT examination in assessing lateral neck lymph nodes was only 27%^11^. The number of central lymph node metastasis was often used to predict whether metastasis occurred in clinical practice. Zeng *et al*. suggested to perform lateral neck lymph node dissection when the number of positive central lymph nodes was ≥2^10^. However, Cai *et al*. suggested that central lymph node metastasis in patients with papillary thyroid carcinoma was related to lateral neck metastasis. When the number of central lymph node metastasis was ≥3, it could be used as a quantitative index for predicting lateral neck lymph node metastasis; also, lateral neck lymph node dissection was recommended in this case^29^. So *et al*. analyzed the data of 18,741 patients with papillary thyroid carcinoma in 23 studies. They found that the significant risk factors for lateral lymph node metastasis were not much different from known risk factors for central lymph node metastasis. Extrathyroidal extension, tumor multifocality, male sex, upper pole location, tumor size ≥1.0 cm, lymphovascular invasion and tumor bilaterality were significantly associated with lateral lymph node metastasis^30^. Our study results showed that TSH, metastatic rate of central lymph nodes, thyroid carcinoma combined with Hashimoto’s disease, and number of tumors were the independent risk factors for lateral neck metastasis in patients with thyroid carcinoma. The aforementioned studies predicted lateral neck lymph node metastasis with a single index, which lacked objectivity and comprehensiveness. Therefore, logistic regression model and ROC curve were used in this study to comprehensively analyze the clinicopathological features of patients with multiple papillary thyroid carcinomas. More risk factors were found for lateral neck lymph node metastasis in patients with papillary thyroid carcinoma. Therefore, a prediction model was developed in this study for lateral neck lymph node metastasis in patients with papillary thyroid carcinoma. The results showed that the AUC of the prediction model was the largest (0.783), positive predictive rate was 95.3%, sensitivity was 54.7%, specificity was 93.5%, Youden’s index was 48.2%, and prediction accuracy was 78.3% compared with the single risk factor. This implied that the model had a better predictive ability for lateral neck lymph node metastasis in patients with papillary thyroid carcinoma compared with the single index. In clinical practice, if a high probability of lateral neck lymph node metastasis is detected in the patients by preoperative examination, the patient may undergo a surgery for the resection of lateral neck lymph nodes. However, occasionally, there is lack of evidence for the metastasis in the lateral neck lymph node by preoperative examination but pathological results confirmed the central lymph node metastasis. In this case, whether the lateral neck lymph node should be disrespected is yet to be elucidated. Thus, the present model provided a method to predict whether or not the lateral neck lymph node metastasis occurred in the non-consecutive patients. Moreover, the study aimed to reduce the unnecessary lateral neck lymph node resection, and the patients with lateral neck lymph node metastasis may early undergo reasonable treatment. In conclusion, the model provided a way to predict scientifically, quantitatively, and accurately whether lateral neck lymph node metastasis occurred in patients with papillary thyroid carcinoma. It was highly promising in determining reasonable surgical range, reducing unnecessary lateral neck lymph node dissection, and further improving the quality of life of patients.

## Methods

### Ethics

This study conformed to the Declaration of Helsinki regarding ethical principles for medical research in humans. It was approved by the biomedical ethics committee of Inner Mongolia Medical University (No. YKD2014063).

### General data

A total of 106 initially treating patients with papillary thyroid carcinoma at the thyroid surgery department of the Affiliated Hospital of Inner Mongolia Medical University were enrolled from January 2009 to April 2017. Inclusion criteria were as follows: (1) patients with complete medical records; (2) patients who were operated by the same medical team; (3) patients who were pathologically confirmed as papillary thyroid carcinoma; (4) patients who underwent neck lymph node dissection (including central and lateral neck lymph nodes) at the same time; (5) patients without the combination with neck and other malignant tumors; (6) patients without a previous history of head and neck radiotherapy; and (7) patients without distant metastasis. Surgical procedure: Standardized surgery was performed according to the Chinese version of guidelines for “diagnosing and managing thyroid nodules and differentiated thyroid cancer”^25^. Patients underwent total thyroidectomy, as well as ipsilateral central and lateral neck lymph node dissection. Neck lymph node dissection: (1) central lymph node dissection (level VI): The dissection range was from the thyroid cartilage to the suprasternal fossa. The lateral border was the medial margin of carotid sheath, including anterior tracheal, paratracheal, and anterior laryngeal lymph nodes. (2) Lateral neck lymph node dissection (levels II–V): The dissection range was from digastric muscles to the clavicle. The medial border was the medial margin of the carotid sheath, and the lateral border was up to the lymph node and soft tissue of the anterior border of trapezius. Background information: Data on patient’s age; gender; serum tri-iodothyronine (T3), thyroxine (T4), free triiodothyronine (FT3), free thyroxine (FT4), thyroid-stimulating hormone (TSH), antithyroglobulin antibody (TG-Ab), and thyroid peroxidase antibody (TPO-Ab) levels; size and location of primary lesions; tumor diameter; multifocality; and capsular invasion were collected. The pathological type of primary lesions, whether combined with Hashimoto’s disease, and the status of neck lymph node metastasis were determined based on pathological results.

### Statistical analysis

Microsoft Excel was used to organize data. SPSS 21.0 (SPSS, IL, USA) software was used for data analysis. Enumeration data were presented as the number of cases and composition ratio. Measurement data were presented as mean ± standard deviation. The χ² test, *t* test, and Fisher’s exact test were used for single-factor analysis. Nonconditional logistic regression was used for the multivariate analysis of lateral neck lymph node metastasis in patients with papillary thyroid carcinoma for the variables with *P* < 0.10. The stepwise regression method was used for selecting independent variables. After ROC curve was established, the area under the curve (AUC) was calculated. The inspection level *α* = 0.05, and the *P* value ≤ 0.05 was considered statistically significant. A model for predicting lateral neck lymph node metastasis in patients with papillary thyroid carcinoma was developed based on the results of statistical analysis.

## Electronic supplementary material

Dataset 1
