# Supplementary material for: Establishing a prediction model for lateral neck lymph node metastasis in patients with papillary thyroid carcinoma
Source: Sci Rep. 2018 Nov 26;8:17355. doi: 10.1038/s41598-018-35551-9 (PMC6255803; doi:10.1038/s41598-018-35551-9)
Supplement: Supplementary file 1 — Dataset 1 [file 41598_2018_35551_MOESM1_ESM.pdf]

Establishing a prediction model for lateral neck lymph node metastasis in patients with papillary thyroid carcinoma

Shan Jin, Wuyuntu Bao, Yun-Tian Yang, Tala Bai, Yinbao Bai

Department of General Surgery, Affiliated Hospital of Inner Mongolia Medical University, Hohhot 010050, Inner Mongolia Autonomous Region, China.

Correspondence to:

Shan Jin, MD, PhD,

Department of General Surgery,

Affiliated Hospital of Inner Mongolia Medical University,

Tongdao North Rd 1, Hohhot 010050,

Inner Mongolia Autonomous Region, China;

Tel: +86-471-3451465

Fax: +86-471-3451465

E-mail: jinshangood@163.com

```
LOGISTIC REGRESSION VARIABLES lateral_neck_lymph_node_metastasis
  /METHOD=FSTEP(LR) TSH TGAb Hashimoto's_disease Capsular_invasion
No._of_tumors Multifocality No._of_central_lymph_node_metastasis
Metastatic_rate_of_central_lymph_nodes
  /CONTRAST (Hashimoto's_disease)=Indicator(1)
  /CONTRAST (Capsular_invasion)=Indicator(1)
  /CONTRAST (Multifocality)=Indicator(1)
  /PRINT=CI(95)
  /CRITERIA=PIN(0.05) POUT(0.10) ITERATE(20) CUT(0.5).
```

Logistic Regression

Notes

Output Created		10-AUG-2018 18:04:27
Comments		
Input	Data	D:\jinshan （8.10）.sav
	Active Dataset	DataSet1
	Filter	<none>
	Weight	<none>
	Split File	<none>
	N of Rows in Working Data File	106
Missing Value Handling	Definition of Missing	User-defined missing values are treated as missing
Syntax		LOGISTIC REGRESSION VARIABLES lateral_neck_lymph_node_metastasis /METHOD=FSTEP(LR) TSH TGAb Hashimoto's_disease Capsular_invasion No._of_tumors Multifocality No._of_central_lymph_node_metastasis Metastatic_rate_of_central_lymph_nodes /CONTRAST (Hashimoto's_disease)=Indicator(1) /CONTRAST (Capsular_invasion)=Indicator(1) /CONTRAST (Multifocality)=Indicator(1) /PRINT=CI(95) /CRITERIA=PIN(0.05) POUT(0.10) ITERATE(20) CUT(0.5).
Resources	Processor Time	00:00:00.05
	Elapsed Time	00:00:00.03

Case Processing Summary

Unweighted Cases[a]		N	Percent
Selected Cases	Included in Analysis	83	78.3
	Missing Cases	23	21.7
	Total	106	100.0
Unselected Cases		0	.0
Total		106	100.0

a. If weight is in effect, see classification table for the total number of cases.

Dependent Variable Encoding

Original Value	Internal Value
no	0
yes	1

Categorical Variables Codings

		Frequency	Parameter coding (1)
Multifocality	no	50	.000
	yes	33	1.000
Capsular_invasion	.00	64	.000
	1.00	19	1.000
Hashimoto’s_disease	no	55	.000
	yes	28	1.000

Block 0: Beginning Block

Classification Table[a,b]

Observed			Predicted: lateral_neck_lymph_node_metastasis no	Predicted: lateral_neck_lymph_node_metastasis yes	Percentage Correct
Step 0	lateral_neck_lymph_node_metastasis	no	0	28	.0
		yes	0	55	100.0
	Overall Percentage				66.3

a. Constant is included in the model.

b. The cut value is .500

Variables in the Equation

		B	S.E.	Wald	df	Sig.	Exp(B)
Step 0	Constant	.675	.232	8.457	1	.004	1.964

Variables not in the Equation

			Score	df	Sig.
Step 0	Variables	TSH	4.994	1	.025
		TGAb	2.835	1	.092
		Hashimoto's_disease(1)	2.863	1	.091
		Capsular_invasion(1)	3.550	1	.060
		No._of_tumors	7.507	1	.006
		Multifocality(1)	5.928	1	.015
		No._of_central_lymph_node_metastasis	8.695	1	.003
		Metastatic_rate_of_central_lymph_nodes	14.147	1	.000
	Overall Statistics		30.608	8	.000

Block 1: Method = Forward Stepwise (Likelihood Ratio)

Omnibus Tests of Model Coefficients

		Chi-square	df	Sig.
Step 1	Step	14.558	1	.000
	Block	14.558	1	.000
	Model	14.558	1	.000
Step 2	Step	8.329	1	.004
	Block	22.888	2	.000
	Model	22.888	2	.000
Step 3	Step	8.899	1	.003
	Block	31.787	3	.000
	Model	31.787	3	.000
Step 4	Step	5.054	1	.025
	Block	36.841	4	.000
	Model	36.841	4	.000

Model Summary

Step	-2 Log likelihood	Cox & Snell R Square	Nagelkerke R Square
1	91.559[a]	.161	.223
2	83.230[b]	.241	.334
3	74.331[c]	.318	.441
4	69.277[c]	.358	.497

a. Estimation terminated at iteration number 4 because parameter estimates changed by less than .001.

b. Estimation terminated at iteration number 5 because parameter estimates changed by less than .001.

c. Estimation terminated at iteration number 6 because parameter estimates changed by less than .001.

Classification Table[a]

Observed			Predicted: lateral_neck_lymph_node_metastasis no	Predicted: lateral_neck_lymph_node_metastasis yes	Percentage Correct
Step 1	lateral_neck_lymph_node_metastasis	no	13	15	46.4
		yes	11	44	80.0
	Overall Percentage				68.7
Step 2	lateral_neck_lymph_node_metastasis	no	16	12	57.1
		yes	8	47	85.5
	Overall Percentage				75.9
Step 3	lateral_neck_lymph_node_metastasis	no	17	11	60.7
		yes	6	49	89.1
	Overall Percentage				79.5
Step 4	lateral_neck_lymph_node_metastasis	no	18	10	64.3
		yes	7	48	87.3
	Overall Percentage				79.5

a. The cut value is .500

Variables in the Equation

		B	S.E.	Wald	df	Sig.
Step 1[a]	Metastatic_rate_of_central_lymph_nodes	.285	.081	12.562	1	.000
	Constant	-.945	.497	3.617	1	.057
Step 2[b]	No._of_tumors	1.237	.499	6.132	1	.013
	Metastatic_rate_of_central_lymph_nodes	.290	.087	10.988	1	.001
	Constant	-2.709	.906	8.939	1	.003
Step 3[c]	TSH	.588	.235	6.274	1	.012
	No._of_tumors	1.404	.568	6.115	1	.013
	Metastatic_rate_of_central_lymph_nodes	.334	.097	11.888	1	.001
	Constant	-4.791	1.331	12.950	1	.000
Step 4[d]	TSH	.681	.248	7.514	1	.006
	Hashimoto's_disease(1)	1.463	.691	4.486	1	.034
	No._of_tumors	1.525	.594	6.584	1	.010
	Metastatic_rate_of_central_lymph_nodes	.342	.101	11.399	1	.001
	Constant	-5.699	1.514	14.168	1	.000

Variables in the Equation

			95% C.I.for EXP(B)	
		Exp(B)	Lower	Upper
Step 1[a]	Metastatic_rate_of_central_lymph_nodes	1.330	1.136	1.558
	Constant	.389		
Step 2[b]	No._of_tumors	3.444	1.294	9.163
	Metastatic_rate_of_central_lymph_nodes	1.336	1.126	1.586
	Constant	.067		
Step 3[c]	TSH	1.800	1.136	2.852
	No._of_tumors	4.071	1.338	12.384
	Metastatic_rate_of_central_lymph_nodes	1.396	1.155	1.688
	Constant	.008		
Step 4[d]	TSH	1.975	1.214	3.213
	Hashimoto's_disease(1)	4.319	1.115	16.725
	No._of_tumors	4.595	1.434	14.729
	Metastatic_rate_of_central_lymph_nodes	1.407	1.154	1.716
	Constant	.003		

a. Variable(s) entered on step 1: Metastatic_rate_of_central_lymph_nodes.

b. Variable(s) entered on step 2: No._of_tumors.

c. Variable(s) entered on step 3: TSH.

d. Variable(s) entered on step 4: Hashimoto's_disease.

Model if Term Removed

Variable		Model Log Likelihood	Change in -2 Log Likelihood	df	Sig. of the Change
Step 1	Metastatic_rate_of_central_lymph_nodes	-53.059	14.558	1	.000
Step 2	No._of_tumors	-45.780	8.329	1	.004
	Metastatic_rate_of_central_lymph_nodes	-48.060	12.891	1	.000
Step 3	TSH	-41.615	8.899	1	.003
	No._of_tumors	-41.566	8.801	1	.003
	Metastatic_rate_of_central_lymph_nodes	-44.524	14.717	1	.000
Step 4	TSH	-40.106	10.936	1	.001
	Hashimoto's_disease	-37.165	5.054	1	.025
	No._of_tumors	-39.497	9.718	1	.002
	Metastatic_rate_of_central_lymph_nodes	-41.748	14.220	1	.000

Variables not in the Equation

			Score	df	Sig.
Step 1	Variables	TSH	6.071	1	.014
		TGAb	1.816	1	.178
		Hashimoto’s_disease(1)	2.709	1	.100
		Capsular_invasion(1)	2.651	1	.103
		No._of_tumors	6.368	1	.012
		Multifocality(1)	5.203	1	.023
		No._of_central_lymph_node_metastasis	.663	1	.415
	Overall Statistics		19.486	7	.007
Step 2	Variables	TSH	6.680	1	.010
		TGAb	1.163	1	.281
		Hashimoto’s_disease(1)	2.947	1	.086
		Capsular_invasion(1)	.608	1	.436
		Multifocality(1)	1.343	1	.247
		No._of_central_lymph_node_metastasis	1.888	1	.169
	Overall Statistics		13.877	6	.031
Step 3	Variables	TGAb	.905	1	.341
		Hashimoto’s_disease(1)	4.824	1	.028
		Capsular_invasion(1)	.323	1	.570
		Multifocality(1)	.687	1	.407
		No._of_central_lymph_node_metastasis	2.360	1	.124
	Overall Statistics		7.754	5	.170
Step 4	Variables	TGAb	.015	1	.902
		Capsular_invasion(1)	.481	1	.488
		Multifocality(1)	.734	1	.391
		No._of_central_lymph_node_metastasis	2.370	1	.124
	Overall Statistics		3.242	4	.518

Model each variable:

Logistic(P)= $-5.699+0.681\times_{(\text{TSH})}+0.342\times_{(\text{Metastatic rate of central lymph nodes})}+1.463\times_{(\text{Combined with Hashimoto's disease})}+1.525\times_{(\text{No. of tumors})}$

TSH: laboratory examination is expressed in μIU/m;
Metastatic rate of central lymph nodes=No. of central lymph node metastasis/ No. of dissection central lymph node;
Combined with Hashimoto's disease: Yes = 1, No = 0;
No. of tumors: the number of pathologically confirmed;
No. of central lymph node metastasis: the number of pathologically confirmed.

If logistic (*P*) was ≥0.821, it was predicted that lateral neck lymph node metastasis occurred in patients with papillary thyroid carcinoma. If logistic (*P*) was <0.821, it was predicted that no metastasis was found in the lateral neck lymph node.
